# Spectrum of heart failure in sub-Saharan Africa: data from a tertiary hospital-based registry in the eastern center of Burkina Faso

**DOI:** 10.11604/pamj.2020.36.30.19321

**Published:** 2020-05-21

**Authors:** Dakaboué Germain Mandi, Joel Bamouni, Rélwendé Aristide Yaméogo, Dangwé Temoua Naïbé, Elisé Kaboré, Yibar Kambiré, Koudougou Jonas Kologo, Georges Rosario Christian Millogo, Patrice Zabsonré

**Affiliations:** 1 Cardiology Unit, Department of General Medicine, Regional Hospital Center of Tenkodogo, Tenkodogo, Burkina Faso; 2 Superior School of Health Sciences, University of Ouahigouya, Ouahigouya, Burkina Faso; 3 University of Normandie, UNIHAVRE- UNIROUEN - UNICAEN CNRS, UMR IDEES, Le Havre, France; 4 Faculty of Human Health Sciences, University of N’Djamena, N’Djamena, Chad; 5 Training and Research Unit of Health Sciences, University Professor Joseph Ki-Zerbo, Ouagadougou, Burkina Faso; 6 Department of Cardiology, Teaching Hospital of Yalgado Ouedraogo, Ouagadougou, Burkina Faso

**Keywords:** Heart failure, epidemiology, mortality, morbidity, Africa

## Abstract

**Introduction:**

Heart failure (HF) is a strong contributor to non-communicable diseases burden in sub-Saharan Africa (SSA). Few studies have addressed the pattern of HF in Burkina Faso.

**Methods:**

We conducted a prospective cohort study in patients with acute HF in the Regional Hospital Center of Tenkodogo, eastern region of Burkina Faso. Patients were consecutively enrolled from 1^st^ January 2015 to 31^st^ December 2016 and followed up until June 2017. Primary outcome of interest was mortality.

**Results:**

Overall 318 of 1805 cardiac cases presented with acute HF (17.62 %). Of the 298 patients included in the analysis process, 239 had de novo HF and 150 were male. The mean age was 58.56 ± 18.54 years. Eighty-eight patients presented with atrial fibrillation. The mean left ventricular ejection fraction (LVEF) was 38.20 ± 12.85 % with reduced ejection fraction (LVEF < 40%) accounting for 59.73% of the cases. Most of the study patients lived in rural areas. Hypertensive heart disease (50.34%) and idiopathic dilated cardiomyopathy (19.80%) were the leading causes of HF. Most patients received renin-angiotensin system blockers contrasting with a lower prescription rate of beta-blockers (99% versus 18.79% respectively). The incidence of all-cause mortality was 31 percent patients-years.

**Conclusion:**

Heart failure is frequent in SSA, affecting patients at younger age. Predominantly of non-ischemic cause, commonly hypertensive, the disease is associated with high mortality.

## Introduction

Heart failure (HF) is a major public health and growing problem, affecting about 26 million people worldwide [1] including low-income countries particularly sub-Saharan Africa (SSA) [2,3]. The prevalence of HF is approximately 1-2% of the adult population in developed countries with well-established population-based registries, rising to ≥ 10% among people over 70 years of age [2-5]. This prevalence is going to scale up because of the ageing population and improvements in treatment [6]. HF is associated with high morbidity and mortality despite progress in its management. Expenditure of HF-related health care is estimated to be about US$ 100 billion in 2012 [7], with total costs expected to strongly increase in the forthcoming decades [6]. Data on HF in sub-Saharan Africa (SSA) are still scarce and mostly available for urban hospital-based settings [8]. Therefore, the present study aimed to assess the clinical epidemiology and long-term prognosis of HF in the eastern center region of Burkina Faso, West Africa.

## Methods

**Study setting:** this study was conducted in the cardiology unit, Department of Medicine, Regional Hospital Center (RHC) of Tenkodogo, Tenkodogo, Burkina Faso. The RHC of Tenkodogo is the unique tertiary health care center that covers a dry orchard savannah region populated of about 1.4 million inhabitants, almost constituted by subsistence farmers. Tenkodogo, the capital town of the eastern-center region is located at 188 kilometers from the political capital, Ouagadougou. Since the opening of the RHC, the first cardiologist was assigned in 2015.

**Procedures:** recruitment was performed in consecutive patients ≥ 18 years old with acute heart failure (AHF) who attended the cardiology unit of the RHC between 1^st^ January 2015 and 31^st^ December 2016. The diagnosis of AHF was based on the European Society of Cardiology Guidelines criteria [9]. AHF was thereafter classified into new-onset (de novo) AHF as well as acutely decompensated chronic heart failure (ADCHF). New-onset AHF referred to patients with no prior history of HF. ADCHF was defined as worsening of HF in patients with a previous diagnosis or hospitalization for HF. Baseline data were collected on discharge from hospital (inpatients) and on one-week visit after onset of AHF treatment (outpatients) through a standardized case report form using both inpatients and outpatients´ registries. Sociodemographic data (age, gender, residence) were recorded. History of hypertension, mellitus diabetes, dyslipidemia, smoking, alcohol abuse was collected. New York heart association (NYHA) functional class, weight and height were assessed. Patient´s serum creatinine, sodium, glucose, uric acid and hemoglobin were measured. Thyroid hormones were checked when clinically relevant. A 12-lead resting electrocardiogram (ECG) was recorded for each patient at baseline. Trans-thoracic echocardiography was performed for the diagnosis of underlying heart diseases according to American society of echocardiography (ASE) guidelines. Left ventricular ejection fraction (LVEF) was measured using Simpson´s biplane method [10]. HF was afterwards classified with preserved (HFpEF), mid-range (HFmrEF) and reduced ejection fraction (HFrEF) [11]. Kidney function was assessed using the chronic kidney disease epidemiology collaboration (CKD-EPI) creatinine equation [12]. Medications prescribed to study patients on discharge from hospitalization or on day 7 visit (outpatients) were also reported as baseline treatment.

**Operational definitions:** kidney dysfunction was defined as an estimated glomerular filtration rate (eGFR) < 60 ml/min/1.73 m^2^. Corrected QT interval was reported by measuring manually the RR and QT intervals from the 12-lead ECG. Thereafter, Bazett's formula (QTc = QT/√RR) was applied for correction of QT intervals for heart rate [13]. The diagnosis of hypertensive heart disease was based on the presence of echocardiogram abnormalities such as left ventricular hypertrophy (concentric or eccentric), increased left ventricular mass index, enlarged left ventricular or left atrial size and increased volumes and diastolic or systolic left ventricular dysfunction in patients with hypertension apart from obvious alternative explanation. Ischemic heart disease was diagnosed in patients with either history of typical angina or acute myocardial infarction and/or typical ECG abnormalities of acute myocardial infarction or myocardial ischemia, associated with ventricular regional wall motion abnormality on 2D echocardiography. Valvular heart disease diagnosis was based on joint ESC/EACTS guidelines on the management of valvular heart disease [14]. Restrictive cardiomyopathy was considered in the presence of normal or reduced diastolic volumes (of one or both ventricles), normal or reduced systolic volumes and normal ventricular wall thickness [15]. Idiopathic dilated cardiomyopathy was considered in the presence of left ventricular dilatation and left ventricular systolic dysfunction in the absence of abnormal loading conditions (hypertension, valve disease), coronary artery disease, congenital heart disease or any obvious clinical condition sufficient to cause global systolic impairment. Right ventricular dilatation and dysfunction may be present but are not necessary for the diagnosis [15]. Peripartum cardiomyopathy was considered in the presence of symptoms of congestive heart failure developed in the last month of pregnancy or during the first five months post-partum, left ventricular ejection fraction ≤ 45% by transthoracic echocardiography, in sinus rhythm without identifiable cause of heart failure. Myocarditis was considered in heart failure patients according to 2013 European society of cardiology diagnostic criteria for clinically suspected myocarditis [16], as cardiac magnetic resonance imaging, histological, immunological and immunohistochemical criteria were not available at the study site.

**Follow-up:** patients were followed up until 30^th^ June 2017. Additionally, telephone contact was used to ascertain the survival status from patients or their relatives as national mortality registry is so far not available countrywide.

**Outcomes:** the primary outcome of interest was all-cause mortality through follow-up period. Subsequently, readmission for acute decompensation of HF and stroke were assessed. The immediate cause of death was also recorded and categorized as either cardiac, non-cardiac or unknown.

**Statistical analysis:** data were analyzed using R software. Continuous variables were expressed as means ± standard deviation (SD) and categorical variables as percentage. Differences between groups were assessed using Chi square or student test as appropriate. Survival rates over time were provided using the Kaplan-Meier method. A value of p < 0.05 was considered statistically significant.

**Ethic aspects:** the study was approved by the institution´s ethics review board. The board waived the need for signed written informed consent due to illiteracy of most study participants and also that collected data were non-invasive and obtained from routine practice. Only oral informed consent was required. The study was carried out in accordance with the principles of the declaration of Helsinki [17].

## Results

Overall, 1805 patients attended the cardiology unit during the recruitment period, (both outpatients and inpatients). AHF was reported in 318 patients accounting for 17.62% of all admissions. Twenty patients were excluded from the final analysis process (in-hospital death before enrolment: 10, uncomplete data: 3, moved to neighboring countries: 3 and lost to follow-up: 4). Of the remaining 298 patients (205 in-patients, 93 outpatients), 239 were de novo HF cases and 150 patients were male. The mean age was 58.56 ± 18.54 years (extremes: 18 - 99 years). Most of the study patients (80.54%) lived in rural areas. Hypertension was the most prevalent cardiovascular risk factor (56.38%). Patients´ baseline epidemiological characteristics are shown in Table 1. Atrial fibrillation was the most common arrhythmia recorded in 88 patients (29.53%). One hundred and seventy-eight patients (59.73%) presented with kidney dysfunction. HFrEF was found in 178 patients. Table 2 shows baseline laboratory findings of the study patients.

**Table 1:** Baseline epidemiological characteristics of all 298 study patients

Characteristic	Values
Number of patients	298
Age (years), mean ±SD	58.56±18.54
Male, n (%)	150 (50.34)
Rural residence	240 (80.54)
Systolic blood pressure (mmHg), mean±SD	131.04±30
Diastolic blood pressure (mmHg), mean±SD	78.86±16.72
**NYHA functional class, n (%)**	
I-II	215 (72.15)
III-IV	83 (27.85)
Heart rate (beats/min), mean±SD	98.67±21.11
**History of heart failure, n (%)**	
Acute decompensation of chronic heart failure	60 (20.13)
New-onset heart failure	238 (79.87)
Delay in attending cardiac unit (days), mean±SD	24.92±12
**Cardiovascular risk factors, n (%)**	
Hypertension	168 (56.38)
Diabetes mellitus	13 (4.36)
Smoking	53 (17.79)
Dyslipidemia	12 (4.03)
Alcohol abuse, n (%)	14 (4.70)

SD, standard deviation; NYHA, New York Heart Association

**Table 2:** Baseline laboratory findings in all 298 patients with heart failure

Characteristic	Values
Cardiothoracic index (mean±SD)	0.66±0.04
Atrial fibrillation, n (%)	88 (29.53)
Atrial flutter, n (%)	1 (0.34)
Premature ventricular complex, n (%)	54 (18.12)
QRS duration (milliseconds), mean±SD	109.06±8.62
Left bundle branch block, n (%)	24 (8.05)
Right bundle branch block, n (%)	8 (2.68)
QTc (milliseconds) mean±SD	441.51±62.21
**Blood sampling, mean±SD**	
Hemoglobin (g/dl)	11.78±2.13
Serum glucose (mmol/l)	4.67±1.13
Sodium (mmol/l)	136.81±7.29
Uric acid (μmol/l)	359.53±104.97
eGFR (ml/min/1.73 m^2^)	65.98±27.48
Kidney dysfunction, n (%)	114 (38.26)
Left ventricular end-diastolic diameter (mm), mean±SD	61.73±8.03
Left ventricular ejection fraction, mean±SD	38.20±12.85
**Heart failure classification, n (%)**	
HFpEF	45 (15.10)
HFmrEF	75 (25.17)
HFrEF	178 (59.73)
TAPSE (mm) mean±SD	15.16±4.58

QTc, corrected QT interval; eGFR, estimated glomerular filtration rate using CKD-EPI equation; HFpEF, heart failure with preserved ejection fraction, HFmrEF, mid-range ejection fraction; HFrEF, reduced ejection fraction; TAPSE, tricuspid annulus plane systolic excursion

Etiologies of HF were dominated by hypertensive heart disease (50.34%) and idiopathic dilated cardiomyopathy (19.80%). Ischemic heart disease was found in 13 patients (4.36%) and valvular heart disease (VHD) comprising both rheumatic (n = 16) and degenerative (n = 4) was reported in 6.71% of the cases. In this report, patients were given angiotensin-converting enzyme-inhibitors/angiotensin receptor blockers (ACEI/ARB), beta-blockers and aldosterone antagonists in respectively 99%, 18.79% and 68.79% of the cases. Etiologies and medications prescribed to study patients are shown in Table 3.

**Table 3:** Etiologies of heart failure and baseline medications prescribed in study patients

Characteristic	Values
Etiologies of heart failure, n (%)	
Ischemic heart disease	13 (4.36)
Hypertensive heart disease	150 (50.34)
Peripartum cardiomyopathy	32 (10.74)
Restrictive cardiomyopathy	17 (5.70)
Valvular heart disease	20 (6.71)
Myocarditis‡	4 (1.34)
Cardiothyreosis	3 (1.01)
Idiopathic dilated cardiomyopathy	59 (19.80)
Medications, n (%)	
ACEI/ARBs	295 (98.99)
Beta-blockers	56 (18.79)
Aldosterone receptor antagonists	205 (68.79)
Loop diuretics*	290 (97.32)
Thiazide	41 (13.76)
Calcium channel blockers	15 (5.03)
Nitrates*	176 (59.06)
Amiodarone	33 (11.07)
Digoxin	88 (29.53)
Vitamin K antagonists	04 (1.34)
Antiplatelet	193 (64.77)
Statins	10 (3.36)
Iron supplementation	65 (21.81)

‡ Including 3 cases of HIV-related and one case of influenza virus, ACEI/ARB: Angiotensin-converting enzyme-inhibitors/Angiotensin receptor blockers

* Loop diuretics and nitrates were only used until complete relief of congestive signs/symptoms.

**Figure 1:**
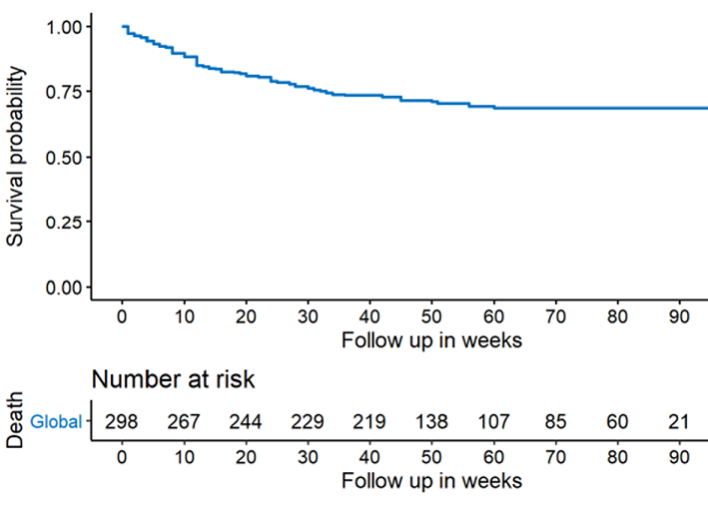
Kaplan-Meier estimates of the cumulative risk for all-cause death in patients with heart failure

The overall patients´ follow up period was 14899 weeks with a mean of 50 ± 27 weeks (extremes: 1-99 weeks). Eighty-nine patients died corresponding to an incidence of 31 percent patients-years. Seventy-seven patients were readmitted for acute decompensation of heart failure and five patients experienced ischemic stroke. Kaplan-Meier estimates of the cumulative risk for all-cause mortality are shown in Figure 1. Deceased patients were ten-years older than those who survived (mean age: 65.37 ± 17.24 years versus 55.66 ± 18.36 years; p < 0.001). The all-cause mortality rate was higher in HFrEF compared to HFmrEF and/or HFpEF (45% versus 7.5% respectively; RR: 1.68, 95% CI: 1.46 - 1.94, p < 0.001). Among patients readmitted for acute decompensation of HF, the overall mortality rate was 57.14% compared to 20.36% in those with readmission-free (RR: 1.86, 95% CI: 1.42 - 2.43, p < 0.001). Death was of cardiac origin in 80 cases including ischemic stroke (n = 2), life-threatening ventricular arrhythmia (n = 6), worsening of acutely decompensated heart failure (n = 5) and sudden death defined as death that occurred unexpectedly in an otherwise stable patient within one hour of the onset of symptoms (n = 67). Non-cardiac cause of death involved sepsis (n = 4) and lung cancer (n =1). In four case, the cause of death was unknown.

## Discussion

This current work is the first one to provide detailed data on a hospital-based pattern of HF in eastern center Burkina Faso. AHF was found to account for 17.62% of all cardiac issues received at the cardiac unit of the RHC. The mean age of patients was 58.56 years. Consistently, previous studies from SSA have reported that HF affects younger and middle-aged individuals [18-24]. In contrast, AHF patients from high-income countries registries are almost 20 years older on average than similar patients from SSA [25-28], suggesting that HF occurs in patients in the prime of their lives in sub-Saharan Africa, with major economic implications because it affects the generation of breadwinners and caregivers [29]. In the present report, the leading causes of HF were hypertensive heart disease and idiopathic dilated cardiomyopathy. Similar findings were observed in other parts of SSA [29]. Valvular heart disease mostly rheumatic was reported in 6.71% of our cases. Recent data have shown a disparate prevalence of valvular heart diseases in SSA patients with HF, ranging from 2.4% to 16.6% depending on age cut-off and/or other inclusion criteria[29].

In developed countries ischemic heart disease is currently the most prevalent cause of heart failure. Conversely, in African patients, the diagnosis of ischemic heart disease may have been underestimated, particularly in those with idiopathic dilated cardiomyopathy, because they did not overtly experience myocardial infarction or severe angina. On top of this, none of our HF patients underwent a coronary angiogram to discriminate between ischemic and non-ischemic heart disease [30]. Moreover, postmortem studies revealed that up to 34 to 50% of patients diagnosed with non-ischemic dilated cardiomyopathy have been shown to have significant coronary artery narrowing [31,32].

It is now well established that ACEI/ARB, beta-blockers and aldosterone receptor antagonists are the cornerstone of the pharmacological treatment that strongly reduce mortality and morbidity in patients with HF [11]. Our patients have benefited from these treatments with markedly low prescription rate of beta-blockers (18.8%). Data on the medications used to treat heart failure in SSA have shown a prescription rate of ACEI/ARB, beta-lockers and aldosterone antagonists in 75.5%, 31.4% and 51.5% of the cases respectively [8]. Discrepancies in prescription rates between medications in our study may be partly explained by the unavailability of beta-blockers in public pharmacies but only in private ones as branded. Hence, they got expensive. Moreover, it has been reported that cardiovascular medicines were available in only 25% of urban and 3% rural communities in low-income countries. These medicines were unaffordable for 60% of low-income countries [33]. Access to essential cardiovascular medicines is also limited and requires a renewed focus by the international community to ensure that appropriate medications are readily available and affordable, similar to that which has been implemented for HIV and malaria in Africa [34]. Subsequently, physicians´ compliance with established guidelines on the management of HF should be noted.

The present report exhibited a high incidence of all-cause mortality at one-year. This observation is consistent with most of the recent findings from SSA [8,29]. In western countries, development in treatments and their implementation have improved survival and reduced the hospitalization rate in patients with HFrEF, although the outcome often remains unsatisfactory [11,35-37]. Overall, the burden of HF remains high worldwide despite large differences in patient characteristics (more younger African patients, causes of HF), suggesting that once HF occurs, it may have a distinct course independent of patient characteristics [29].

Our study is limited by its monocentric aspect; hence data extrapolation nationwide has weakened. Definitely, only patients from “privileged” background who could afford cardiology care and/or those with overt HF attended the cardiology unit and then were included. Therefore, patients with less symptomatic cardiovascular diseases or asymptomatic HF in non-cardiac units and in the community, may have been excluded.

## Conclusion

The prevalence of heart failure is substantial in our setting. It affects patients at younger age with hypertensive heart disease being the most common cause. The disease is associated with poor long-term outcome. We must continue to strike for the reduction of the burden of HF through early detection and adequate treatment of its modifiable risk factors (eg. hypertension, smoking, rheumatic fever) at the community level. A population-based study is needed to better characterize the scope of HF in this community.

### What is known about this topic

- HF is an emerging public health concern in SSA with hypertension being the most prevalent risk factor, mostly in urban areas;
- HF is associated with high long-term mortality.

### What this study adds

- HF is not rare even in less urbanized regions of SSA;
- The pattern of the disease is burdened with the lack of low-cost specific medications in a context of poverty;
- Therefore, policymakers should set-up HF prevention strategies and make available essential drugs for cardiovascular treatment in public health pharmacies nationwide.

## Competing interests

The authors declare no competing interests.
